# People’s Medical Gazette

**Published:** 1853-11

**Authors:** 


ART. IV.—*People's Medical Gazette.* Edited by JOHN DAVIS, M. D., Practicing Physician, Abbeville C. H., S. C.

Here is another added to the list of medical periodicals. As its name implies, it is destined for the fructification, not only of the profession, but of that great world of ignorance about us yclept the public. Certainly a praiseworthy undertaking.

We hardly know how to welcome such an addition to our exchanges. Its object is most laudable if well carried out. But what does the editor intend? Will he merely enlighten the people as to the propriety and justice of setting a very high value on their physicians? or will he lower the standard of professional dignity, by making "every man his own doctor?" We fear that something of this latter sort is to be presented. In this present number, eight pages suffice to present a succinct history of the symptoms, tendencies, and treatment of remittent fever; worms; croup; gout; apoplexy; debility of the stomach; torpor of the bowels; nightmare; *a general* treatise on the management of children; besides sundry recipes for cough pills, digestive pills, chronic rheumatism, and salivation. We have not perused all these eight pages closely enough to decide whether there has been any sacrifice of amplitude to conciseness. If there has not, we envy Dr. Davis his happy manner of saying much in little.

All our medical cotemporaries have cordially welcomed the People's Gazette to our fraternity. We are loth to hold back, when all others extend the hand, and accordingly we felicitate our *nouveau né* editor, and hope sincerely he may have a good time of it. If a year hence he shall find some fond hopes dissipated, and quackery still abroad in the land, let him console himself with the thought that he is not the first to fall in this struggle, and that in the expressive motto which adorns his cover:

"To know thyself, and thy relations to the external world, is to be wise in great things." S. B. H.

